# Practical tips by peer support in chronic vestibular hypofunction: an exploratory survey

**DOI:** 10.3389/fneur.2023.1334038

**Published:** 2024-01-03

**Authors:** Bernd Lode Vermorken, Anna C. Schouten, Lien van Laer, Alonda van Toor, Elke M. J. Devocht, Raymond van de Berg

**Affiliations:** ^1^Department of Otorhinolaryngology and Head and Neck Surgery, Division of Balance Disorders, School for Mental Health and Neuroscience (MHENS), Maastricht University Medical Centre, Maastricht, Netherlands; ^2^Center for Social and Cultural Psychology, Faculty of Psychology and Educational Sciences, University of Leuven, Leuven, Belgium; ^3^Department of Rehabilitation Sciences and Physiotherapy/ Movant, Faculty of Medicine and Health Science, University of Antwerp, Antwerp, Belgium; ^4^Patient Organization DFNA9, Stichting De negende van, Goor, Netherlands

**Keywords:** peer support, patient advice, vestibular hypofunction, vestibulopathy, DFNA9

## Abstract

**Introduction:**

Patients with chronic vestibular hypofunction typically suffer from dizziness, imbalance and oscillopsia (blurred vision); symptoms that pose challenges to everyday life. Currently, advice on how to deal with such challenges is mainly provided by health care professionals (i.e., ENT-surgeons, neurologists, physiotherapists and psychologists). However, fellow patients with a similar condition and a true appreciation of the lived experiences, are likely to provide valuable support and advice as well. The purpose of this study, therefore, was to collect tips and advice from patients with chronic vestibular hypofunction.

**Methods:**

An exploratory survey was designed to collect tips from fellow chronic vestibular hypofunction patients on how to cope with disease-related challenges in everyday life. The survey was distributed both online and in person. The list of tips was coded and analyzed thematically and deductively, by using the international classification of functioning, disability, and health (ICF) model.

**Results:**

In total, 425 tips were obtained from the 179 participants. Most tips were coded under “environmental factors” (46%) and “activities and participation” (39%). The remaining tips were categorized as “body functions” (15%). No tips were about “body structures.” The participants coped with their daily struggles by investing in assistive products and technology, like adapted bikes, special footwear, walking frames. They described the importance of ensuring minimal light intensity for visibility (i.e., installing light sources in dark places). During activities, participants gave the advice to avoid bumpy roads and obstacles, and highlighted the necessity of adequate visual fixation to maintain balance. To ensure optimal activity, participants emphasized the importance of managing energy and taking sufficient rest.

**Discussion:**

This study gives insight into how patients with chronic vestibular hypofunction cope with everyday struggles due to their symptoms. These tips can expand advice given by healthcare professionals. Knowing that fellow patients experience similar struggles and learned to deal with their struggles in adequate ways, might offer support and help patients focus on possibilities rather than on disabilities. Further research should investigate the effect of sharing tips to see whether improvement in (mental) health can be achieved in patients with chronic vestibular hypofunction.

## 1 Introduction

Vestibular hypofunction is mainly characterized by dizziness, imbalance (unilateral hypofunction) and oscillopsia (bilateral hypofunction) for more than 3 months. The condition can be life changing since patients experience a variety of mental and physical symptoms which interfere with daily activities as well as social life (1–3). For example, due to impaired vestibular function, these patients can experience difficulties in walking or cycling and are tired more quickly. They also feel misunderstood when bystanders perceive their unsteady gait as drunkenness or others trivialize the impact of the vestibular hypofunction on general fatigue (2). Although bilateral vestibulopathy might be considered as the most severe variant, 29–66% of patients suffering from unilateral vestibulopathy experience chronic vestibular symptoms of which dizziness and imbalance are most frequently reported (3). When symptoms persist despite vestibular rehabilitation, patients seek help and advice to deal with the chronic consequences of their condition. Patients therefore reach out to various health care professionals, such as ENT-surgeons, neurologists, physiotherapists and sometimes psychologists. While unequivocally valuable, information and advice from health care providers is currently finite due to the limited therapeutic options. Unfortunately, healing is often not possible. Since learning how to cope with this chronic condition is critical, we propose in this study that tips from fellow patients, or peers, could serve as a valuable supplement.

Peer support in the form of sharing experiences and tips is valuable and beneficial in various ways. First, peers are able to offer support, empathy and validation (4). They have a unique understanding of the day-to-day challenges of the condition since they have gone through similar experiences themselves. Knowing that peers succeeded in coping with the consequences of a chronic condition is likely to result in fewer feelings of isolation, and more hope, reassurance, and confidence (5, 6). Second, the kind of support provided by peers might differ slightly from that of health care professionals. More specifically, tips from peers are more practical, concrete and easy to understand (6–8), as they primarily focus on the lived experience of patients. This might facilitate adjustment to the long-term disabilities associated with vestibular hypofunction in a unique way. For example, minor adjustments in and around the house suggested by others might have a major influence on living comfort. Finally, peer-support provides an opportunity for social comparison, giving patients insight in how well they are themselves dealing with a chronic disease like vestibular hypofunction (9). In summary, peer support might be a valuable addition to the multidisciplinary treatment approach for people with vestibular hypofunction.

Considering their value and benefits, peer support programs are increasingly popular and being deployed for a variety of (chronic) diseases (10–13). This is in keeping with the rise of shared decision making, which challenges the sole autonomy of health care professionals. It emphasizes the expertise of patients as a valuable source of knowledge both in research (i.e., patient participation initiatives) and clinical care (14, 15). The use of online support communities, (OSCs) seems to be widespread among vestibular diagnoses (16). However, at this point, implementation of peer support initiatives in care programs for patients with vestibular hypofunction is limited, both in quantity and content. To our knowledge, there is only one study which investigated an online support community for patients with vestibular disorders (16). The findings of this study suggest that OCS engagement provides emotional support and helps patients to develop coping strategies (16). While patients with vestibular disorders engage in peer support, little is known about *what* they share. We build on and extend this study by documenting simple, practical tips actively given by peers based on their experience in dealing with chronic vestibular symptoms, in a controlled survey format.

## 2 Materials and methods

### 2.1 Exploratory survey distribution

This study was part of a cooperation between the department Otorhinolaryngology and Head & Neck Surgery of Maastricht University Medical Centre + (MUMC+) and a patient organization called ‘*Stichting De negende van’*. This organization represents patients suffering from DFNA9 in both the Netherlands and Belgium. DFNA9 is an autosomal dominant and nonsyndromic disorder that is characterized by progressive sensorineural hearing loss and vestibular pathology (17).

Peer advice on living with vestibular hypofunction was investigated by using a questionnaire designed for this specific purpose (see below). First, the survey was distributed at the 4^th^ Knowledge Day organized by ‘*Stichting De negende van’*, which is an annual event by and for patients suffering from DFNA9 in the Netherlands and Belgium. This Knowledge Day stimulates a valuable interaction between health care professionals and patients with their significant others. Secondly, the survey was distributed online, using SurveyMonkey *(SurveyMonkey Inc., San Mateo, California, United Kingdom)* (18). We made use of single post soliciting via a) e-mail—the e-mail database was provided by ‘*Stichting De negende van’* and consisted of 540 addresses from members who are interested in receiving information—and b) two online support communities on Facebook for patients suffering from vestibular pathology (i.e., *‘Stichting De negende* van’ Facebook page & *‘Eerlijk over evenwicht’* Facebook page). Responses were collected anonymously over the period of one month. No reminder posts were administered, and all collected participant data was self-report.

The exploratory survey instructed participants to give tips that helped them deal with daily struggles resulting from vestibular hypofunction. More specifically, the survey asked patients the following question: “we would like to ask you about the tips and tricks you use to make your daily life more enjoyable and/or safer.” This question had an open answer format, meaning that participants could write down any tip that came to mind, and as many tips they wanted (there was no answer limit). Additionally, we collected demographic data (i.e., age, gender) and basic clinical information (i.e., etiology of vestibular hypofunction, severity of symptoms scored from 0 to 10 and duration of balance symptoms) to contextualize the results. The survey was designed and distributed in Dutch (see Supplementary Appendix SI) for an English translation of the survey). The survey fell under the scope of non-WMO research and was conducted in accordance with the Declaration of Helsinki. No incentives were provided to potential participants.

### 2.2 Data collection and thematic analysis

Participants who reported at least one tip related to chronic vestibular hypofunction were included in the final dataset. If participants gave several tips, these were separated and considered as stand-alone tips that were independently analyzed. This study primarily focused on how patients dealt with chronic, rather than acute, symptoms, as vestibular hypofunction is typically characterized by long-term (> 3 months), chronic symptoms that require different coping strategies (and hence give rise to different tips) than acute symptoms. All tips exclusively describing symptoms with a short onset (i.e., vertigo for seconds, nausea for minutes) or matching an acute or episodic vestibular syndrome, were excluded.

The list of tips was coded and analyzed thematically by two researchers: one medical doctor with experience in the vestibular field (BV) and one physiotherapist with a focus on vestibular rehabilitation (LVL). The two researchers had varying disciplinary backgrounds to allow for a more holistic point of view on the tips. First, an inductive approach was used with repeated rounds of reading and categorizing to derive patterns and recurrent themes across the dataset. The coders figured that recurrent themes derived from open coding closely matched with the predefined categories within the international classification of functioning, disability and health (ICF) model (19). It consists of four overarching constructs—body functions, activities and participation, environmental facts, and body structures—and each construct is in turn broken down by the model into several domains (i.e., communication, domestic life, sensory functions). Since the ICF model is an internationally accepted framework, it facilitated communication and comparison between multiple settings (which is relevant given the close cooperation between the outpatient clinic and physiotherapy in this patient population) (20). The model provides information on individuals’ abilities and limitations, which was interpreted as an adequate reflection of the tips. The patient-centered approach aligned with the ICF’s focus on individuals’ perspectives. Moreover, the tips revealed the multifaceted impact of the disease on everyday life. Categorization by the ICF model ensured that the analysis did not restrict the breadth of these impacts. Therefore, it was agreed between the coders to use the terminology from the ICF model as a deductive approach.

First, every tip was categorized to one of the four overarching ICF-defined constructs. Secondly, every tip was allocated to a predefined domain within this construct (19). For example, the tip “Use two bags or a backpack to carry groceries,” would be categorized under the construct “activities and participation,” which in turn was found to belong to the domain “domestic life.” A tip was allocated to the most specific domain to ensure sufficient differentiation between domains belonging to the same over-arching domain or construct. For example, we differentiated between tips about “energy and drive functions” and “sleep functions,” as opposed to coding for all of these tips as “global mental functions.” All tips were coded by each researcher independently. After a first trial, the application of the ICF model was checked between the two coders and discrepancies were discussed. After the second and final coding session, analyses of both coders were combined. The combined coded data was checked by an independent researcher with extensive experience in thematic analysis (AS). AS resolved any disagreements in coding between coders BV and LVL and verified consistency within and differentiation between domains. Most inconsistencies arose due to the fact that tips could be categorized into multiple domains. Collective decisions were based on the following principles: (1) choose the most specific domain possible (e.g., ‘cognitive flexibility’ instead of the more generic ‘global mental functions’), (2) categorize the underlying meaning behind the tip (e.g., tips including content about daily activities and how to adapt these tasks were categorized into the domain ‘carrying out daily routine’).

## 3 Results

**Figure 1:**
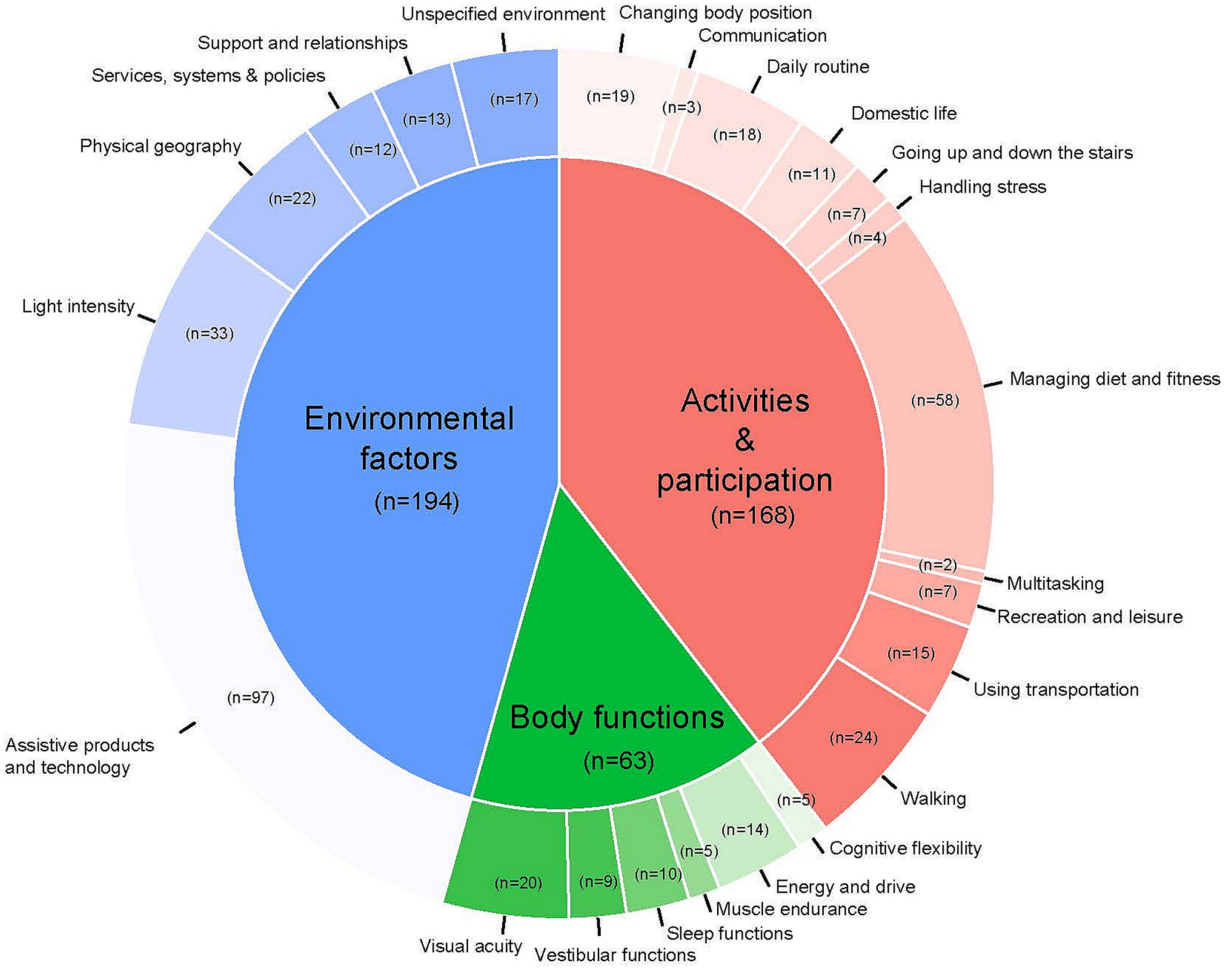
Schematic representation of the relative distribution of each construct and related domains (in alphabetical order) with at least one allocated tip, based on the ICF model (19).

A total of 290 individuals responded to the survey. After inspection 111 individuals were excluded from the analyses because they did not give at least one tip (*n* = 73), or the tips they gave exclusively described symptoms with a short duration (*n* = 38). The remaining 179 participants (66% female) were 60 years old on average (range 17–89) and reported a duration of balance symptoms that varied from one to 60 years (M = 11.6, SD = 10.8). The severity of symptoms ranged from one to ten on the Visual Analog Scale (M = 6.8, SD = 2.3). 122 of the 179 participant filled out the etiology. The distribution of the responses was as follows: 56 DFNA9, 24 unknown, 23 Meniere’s disease, 10 vestibular schwannoma, 6 vestibular neuritis/ labyrinthitis, 3 persistent positional perception dizziness (PPPD). This group generated a total of 425 tips that were included in the final analysis. Figure 1 represents the distribution of the collected tips across constructs and domains. The collected tips are summarized in Table 1, which includes illustrative quotes to represent the findings. No tips were categorized to the fourth construct “body structures.” Domains without allocated tips were not displayed in this study.

**Table 1:** Detailed overview of constructs, domains and their underlying aspects.

Construct	Domain	Specific tip (illustrative quote)	Frequency
Environmental factors			194
	Assistive products and technology		97
		- Consider walking aid: e.g. Nordic walking sticks, rollator	34
		- Consider bike adjustments: e.g. mirror on the bike or trying out a low bike or tricycle	16
		- Install extra bases of support in and around the house: e.g. grab handle in shower, extra banister	14
		- Consider the use of special glasses: e.g. prisma glasses, photogromic sunglasses, night glasses, blue light filter	13
		- Use sturdy flat footwear: e.g. Xsensible shoes	9
		- Consider the use of the BalanceBelt	5
		- Consider a head mounted light	3
		- Consider grip socks	1
		- Consider ear warmers in cold weather conditions	1
		- Use a chair in the shower	1
	Light intensity		33
		- Install (automatic) light sources, especially around the staircase, strips around uneven grounds and in the bedroom	13
		- Make sure there is always light, by taking an extra light source with you	10
		- Planning of activities during day light, try to avoid darkness, especially for driving	7
		- Be careful with bright sunlight and trees, which can cause dizziness. Do not look straight into bright light sources	3
	Physical geography		22
		- Use open, broad and flat roads for walking and cycling	13
		- Remove obstacles as much as possible	3
		- Be careful with leaves, gravel *et cetera*	3
		- Prefer open areas where you can look far away	2
		- Keep a safe distance to the curb	1
	Unspecified environment		17
		- Be aware of your own position in the room: looking at a low-stimulation environment instead of a bright light source, positioning at the end of the room, no turning	5
		- Avoid crowded areas with many stimuli	5
		- No loose objects on the floor, reconsider carpets and cable management. Create structure and orientation	4
		- Install bases of support and seating areas: take care of organizing your furniture, be careful with using multiple colors	3
	Support and relationships		13
		- Clear and honest communication with partner or significant others about your possibilities and limitations, so they will understand the consequences of your hypofunction	6
		- Ask for help, make it specific. People are glad they can help you	4
		- Cancel and reschedule meetings if you think you need it	1
		- Make use of (online) peer-support groups, e.g., use of Facebook groups	1
		- Inform someone at work or your organization about what they can expect from you and your limitations	1
	Services, systems and policies		12
		- Consider a vestibular rehabilitation program	6
		- Consider ergotherapy (for example, Adult Sensory Integration Timmerman Treatment)	4
		- Consider a fall prevention course	2
Activities and participation			168
	Managing diet and fitness		58
		- Stay physically active on a daily base: e.g. by walking / cycling / taking stairs / Ismakogie exercises / working in the garden *et cetera*	54
		- Invest in a healthy diet; aim for structure and variation	4
	Walking		24
		- Use support: e.g. walking hand-in-hand	9
		- Focus at a fixed point far away, like the horizon	4
		- Focus on a straight line on the ground	4
		- Changing your walking pace might be more stable	3
		- A broad gait pattern with your feet rotated outwards might be more stable	2
		- Use applications on your phone to motivate or trigger you with walking activities	2
	Changing and maintaining body position		19
		- Be aware and take your time while making body transitions or head rotations. Consider a micro-pause before transition by looking at a fixed target.	11
		- Trying out different speeds in making body transitions in a safe environment might help you in finding the most optimal speed	2
		- If bending is needed, place one step backward to scan the environment	2
		- Do not turn your head while moving	1
	Carrying out daily routine		18
		- Dosing of tasks and activities; divide and schedule. Rest, regularity and rhythm are very important.	12
		- Try to alternate physical with mental activities and take care of enough resting periods	4
		- Categorizing of tasks in terms of load capacity	1
		- Schedule one day without any plans on a regular base	1
	Moving around using transportation		15
		- Use the front seat of the car	3
		- Make sure you can easily reach the ground with your feet while cycling	3
		- Use a constant tempo while cycling	3
		- Do not cycle in parallel, always behind or in front of someone else	3
		- Do not drive the car in the dusk or dark or with challenging weather conditions	2
		- Be careful with the use of panniers on the bike	1
	Domestic life		11
		- Divide shopping goods/ groceries over two bags or backpack	4
		- Sit down during certain activities, for example while preparing food	2
		- Find support with your foot against the wall while in the bathroom / in standing position	2
		- Plan your shopping within off-peak hours	2
		- Use a bathrobe for drying after showering	1
	Recreation and leisure		7
		- Consider a physical hobby, like Tai Chi / Chi Gong / Yoga	5
		- Consider a hobby with a few other people, but no big groups	2
	Going up and down the stairs		7
		- Always use the banister	5
		- Rotate yourself towards the banister for support and by rotating your feet you can find more support on the staircases	2
	Handling stress		4
		- Try to avoid stress at any time: e.g. by accepting a calmer life style	3
		- Learn a few relaxation and breathing exercises, which you can use whenever needed	1
	Communication		3
		- Use an e-book, so you can adapt spacing *et cetera*	1
		- Use small (mobile) screens, instead of big (computer) screens	1
		- Do not move your head while reading	1
	Carrying out multiple tasks		2
		- Do not multitask while moving around. Take your time for micro activities like sniffing your nose	2
Body functions			63
	Visual acuity functions		20
		- Fixate on an earth fixed target at least a few meters in front of you, instead of looking down while moving around	9
		- Try to avoid visual triggers like busy screens, low sunlight and trees, cars on the highway and flashing images	8
		- Learn how to ‘spot’, fixate gaze after head movement	2
		- Be patient and try not to look at all stimuli at once, divide visual information in pieces	1
	Energy and drive functions		14
		- Schedule enough resting periods, especially after many stimuli	7
		- Take care of a scheduled energy management	3
		- Learn to know your own signals of limitation	2
		- Balance your life with activities and resting periods	2
	Sleep functions		10
		- Respect your sleep, both quality and quantity	8
		- A powernap during the day in bed might be very valuable	2
	Vestibular functions		9
		- Search for proprioceptive input, by holding a finger against the wall while walking for example	5
		- Do not avoid all stimuli that trigger symptoms, try to trigger yourself regularly	3
		- Take time to familiarize yourself with the environment	1
	Muscle endurance		5
		- Consider training programs to increase muscle power and endurance, this might positively influence your balance	5
	Cognitive flexibility		5
		- Learn and accept your limits and possibilities, try to adapt your lifestyle in order to build up new possibilities	2
		- Consider thinking exercises that can help in positive thinking; write down three positive events sometimes, which you can use during down days	2
		- Try to motivate yourself to keep on trying	1

Most of the tips (46%) were categorized under the construct of “environmental factors,” which suggests that many tips referred to or made mention of the physical, attitudinal, and social environment in which patients conduct their lives (see the ICF). Participants’ tips revealed that they make frequent use of a variety of assistive products and technologies to improve their functioning and help them carry out activities. Participants advised to use a walker, wear special footwear, or put on a balance belt (21) to facilitate walking. Others suggested to use adapted bikes (i.e., with mirrors) for cycling. Apart from assistive devices, it was advised to avoid physical geography that makes it harder to keep one’s balance during physical activities, such as bumpy and narrow roads with limited overview. To improve visibility, participants suggested to adjust the light intensity in dark spaces at home, for instance by installing (automated) light sources. While sufficient light is important, participants at the same time pointed out that too much light can hinder functioning and balance. Some of them advised to wear sunglasses or avoid blinking sunlight (i.e., when cycling or on the train). Additionally, participants gave tips about how to change aspects of the home environment other than light, such as freeing the floor of obstacles (cables, carpets), and furnishing the house with little furniture. Finally, participants suggested ways to deal with highly stimulating environments, i.e., by sitting next to the window rather than in the middle of a crowded room. Some participants even advised to avoid busy places altogether. Besides tips to accommodate to or change the physical environment, participants additionally emphasized the importance of managing support and help from their social environment. They advised to communicate openly and honestly with significant others about one’s needs and limitations. Participants also suggested to seek help from health care professionals, such as occupational therapists or vestibular physical therapists (vestibular rehabilitation). Specific courses, such as fall prevention courses and mindfulness were recommended. Taken together, participants mostly shared their experience with adding facilitators and removing barriers in their physical and social environment.

Thirty-nine percent of the tips were categorized under the construct “activities and participation,” which means that a significant part of the tips referred to difficulties experienced while executing activities. Participants primarily mentioned the importance of managing fitness (categorized under the ICF domain “managing diet and fitness”). A significant part of the tips stated that physical activity is key in coping with symptoms; according to participants, patients should aim to keep up with day-to-day activity *despite* symptoms. Walking and cycling were frequently mentioned activities that help participants stay fit. Moreover, the tips that referred to recreation and leisure revealed a preference for activities that improve stamina and ensure physical activity, such as tai chi, qi gong and yoga. General activities like going up and down the stairs, domestic life (e.g., going to the supermarket), or driving a car were mentioned and adapted in such a way that it enabled continuation of these activities. Participants additionally offered tips on *how* to stay physically active: they advised to use visual fixation points, widen the base of support, change the frequency and pace of walking, and use tactile feedback. For example, participants underlined the importance of holding onto the stair banister and positioning one’s feet carefully when taking the stairs. Furthermore, participants specifically shared their ideas related to changing and maintaining body position during these activities. For example, participants paid attention to the speed of head movements and head position. They also advised to fragment transfers (i.e., of the body from one place to another) into multiple phases, and to take time for recalibration in between movements. Finally, participants thought it essential to plan and temporize daily activity to be able to execute tasks. It was suggested that a daily routine helps to avoid stress around activities or tasks. In summary, participants emphasized the importance of staying active and performing daily tasks. Tips were about optimizing, rather than avoiding, such activity.

The final 15% of tips were categorized under the construct “body functions”, suggesting that tips were related to impairments of body functions. Several tips reveal that participants frequently experience a lack of energy. Therefore, they emphasized the importance of taking sufficient rest and getting enough sleep. Maintaining energy levels reduces the burden of symptoms and enables the maintenance of physical fitness, an important category of tips mentioned earlier, as part of the construct ‘activities and participation’. Reported tips revealed that participants often experience symptoms related to their vestibular functions, such as balance issues. They hence gave suggestions for how to stabilize these symptoms, such as using visual (i.e., focus vision on the horizon, no looking down) and proprioceptive information (i.e., touching a wall while walking, holding onto someone). Participants also suggested to do muscle training, such that the body is strong enough to compensate for loss of balance. Apart from body empowerment, cognitive well-being was discussed by providing tips about positive thinking, self-motivation and setting boundaries.

## 4 Discussion

This study aimed to collect practical peer-to-peer tips from patients with vestibular hypofunction. A comprehensive overview of advice on how to deal with symptoms in daily life was established. Tips were collected by means of an exploratory survey and thematically analyzed using the ICF model (19). Most tips focused on changing or adjusting the physical environment (i.e., using assistive products such as special footwear, adapted bikes, and ensuring optimal light intensity), and staying active, albeit in a systematic and balanced way. Finally, participants emphasized the importance of taking rest and managing energy levels to ensure proper body functions. Overall, tips covered three out of four constructs of the ICF model: environmental factors, activities and participation, and body functions. No tips were categorized under body structures. Presumably this finding originates from the fact that this construct is rather technical in nature. It covers the functioning of precise anatomical parts of the body, such as organs, limbs *et cetera*, which most patients have less knowledge and expertise about.

The results were insightful in two ways. First, the results of this study shed light on the challenges patients with vestibular hypofunction face in several life domains. Secondly, the tips gave an idea of how patients deal with these everyday challenges to enhance their quality of life. The peer advice can be helpful for both health care professionals and fellow patients alike.

### 4.1 Insight into everyday challenges experienced by vestibular hypofunction patients

Foremost, these tips show that vestibular patients were able to share practical tips with peers about their personal experiences in multiple domains. The tips given by participants were not restricted to unsteadiness (e.g., losing balance during fast movements) and oscillopsia, but provided insight into a wider range of everyday challenges: situations involving darkness and uneven grounds, cycling, driving a car, dual tasks, and going to the supermarket. Previous studies suggested that stimulation of alternative sensory systems contributes to compensation on the one hand, but a decrease in energy levels and cognitive functioning on the other (22, 23). This was also reflected in the tips. For example, participants mentioned that maintaining a daily routine, managing energy levels and being cognitively flexible were key to dealing with symptoms and the consequences of compensation. Overall, the constructed domains of tips and the situations described were comparable to symptoms cited by patients in previous research, showing the analogy of these results in this specific patient population (2, 3). It is interesting to point out that the relative easiness with which patients translate their symptomatology into tips, appears to be in contrast with the traditional way of history taking which is often challenging and time consuming for both patients and health care provides.

The tips signify that compensation strategies and available vestibular rehabilitation options (24, 25) are unable to eliminate all symptoms associated with the condition of vestibular hypofunction. However, the majority of tips show the absolute importance of staying active. In sum, the peer tips in this study provide illustrative examples and a pragmatic interpretation of the known symptomatology.

### 4.2 The benefits of peer advice for fellow patients and health care professionals

The collected tips in this study could be helpful for both fellow patients and health care professionals. For peers, the tips mainly aim to encourage a shift in focus from avoiding activities and environments due to perceived impairment, to possibilities and opportunities. These tips may help other peers with ‘positive thinking’. It might activate other peers to think of possible ways to overcome challenges and try out new strategies (26). Peer support can improve coping and help patients to maintain their independence by feeling more confident to perform activities. However, further research is needed to evaluate the possible effect of these tips on coping, independence and therefore quality of life in this patient population (27, 28).

The pragmatic tips collected in this study could assist healthcare professionals in giving more specific and actionable recommendations. For instance, these peer-to-peer insights might be presented to future patients by means of an easy-to-read folder in outpatient clinics, which could be designed per symptom category, in order to provide personalized information. These folders could also optimize current patient guides aiming to inform patients on a larger scale. Additionally, tips could potentially be used to adapt professional advice if peers question the efficacy or relevance of the advice. In essence, peer support may play an increasingly pivotal role in the popular shared decision-making model and may serve as a valuable addition to the clinical expertise. Further research is needed to assess the extent and routes to which health care professionals could collaborate with patients, to enhance their treatment plans.

### 4.3 Limitations

This study comes with several limitations. First, by distinguishing between acute and chronic symptoms and by applying a deductive coding approach based on the ICF model, personal tips given by patients were translated into categories defined by health care professionals. This might introduce a professional bias, and comes with the risk of excluding patient perceived symptoms that do not fit the preconceived definition of ‘chronic vestibular syndrome’. Nonetheless, the fact that all tips could be categorized into domains and in some cases even into multiple domains advocates for the applicability of the ICF model.

Second, the tips provided by participants were personal tips that might not be directly applicable to other patients. There are many individual differences in the challenges patients face, and in the strategies that are perceived as helpful in dealing with these challenges. Some ways of dealing with symptoms might be effective for one patient, but not for another patient. It is important that patients use coping strategies that are feasible and fit their individual needs. Health care professionals can play a central role in searching for the right strategies that are personalized to every patient. Moreover, they might evaluate the quality of tips to avoid that unrealistic tips are presented to patients. For example, they could filter tips about certain medication without proven indication and/or efficacy. Taken together, although tips by peers can be a source of inspiration for how to deal with vestibular hypofunction, they cannot be applied indiscriminately to every patient.

Third, while it is encouraging and hopeful to know that others have gone through similar experiences and managed to deal with symptoms caused by vestibular hypofunction, peer support in the form of tips can also be overwhelming and discouraging. Tips from others may arouse feelings of confusion or lead to unrealistic expectations (6). Hope can turn into false hope, or even despair if others seem to be managing better. Patients and healthcare professionals should hence be aware of the potential negative consequences of peer support. Interim quality control by healthcare professionals can reduce patients’ sensation of feeling overwhelmed by the advice they receive. For example, professionals should tailor tips to the needs and capabilities of each patient.

Fourth, since all data was self-reported in a non-clinical setting and not confirmed by a health care professional, it is unclear whether the medical history data (i.e., etiology, duration, severity) provided by participants was accurate. However, it is very plausible that most participants were DFNA9 patients (46% of the reported etiologies) as the in-person and online setting included mostly (family of) members and attendees of the DFNA9 Knowledge day. Consequently, it is also sensible to assume that the majority of participants in our sample, who were on average 60 years old, suffered from significant chronic vestibular hypofunction, given previous findings that most DFNA9 patients older than 60 years old show areflexia (29). Moreover, due to the etiology of DFNA9, it is expected that most participants suffered from a relative slow and progressive form of vestibulopathy, as well as from progressive sensorineural hearing loss, which in theory might influence the tips given by these patients. Categorizing of tips within specific etiologies or degrees of hypofunction was not possible in the current study. However, based on the responses on etiology, it can be assumed that participants mostly suffered from peripheral vestibular disorders. Further research is needed to, for example, differentiate tips between (a) recent and fast progressive versus long-term and slow progressive chronic vestibulopathy, (b) unilateral versus bilateral vestibulopathy patients, and (c) central versus peripheral vestibular disorders.

Lastly, as the link to the survey was publicly accessible, it is unclear how many individuals were exposed to this survey, and whether there were individuals who decided against participation. Consequently, it cannot be ruled out that this study suffered from nonresponse bias or selection bias. Since mobility-impaired individuals were likely underrepresented during the in-person meeting day, it might be possible that the study population was a selection of relatively physically active and well-compensated patients. This might have resulted in an overestimation of the average vestibular hypofunction patient.

## 5 Conclusion

This study collected concrete tips from vestibular hypofunction patients, using an exploratory survey. The practical tips provide illustrative examples and a pragmatic interpretation of the known symptomatology. Tips covered several life domains of the ICF model, and mainly emphasized compensation efforts by optimizing alternative sensory feedback systems. These tips might be a significant source of peer support in patients suffering from chronic vestibular hypofunction. Moreover, the results provide a jumping board for health care professionals to develop and optimize current patient guides aiming to inform patients suffering from chronic vestibular hypofunction.

## Data availability statement

A summary of the original contributions presented in the study are included in the article/Supplementary material, further inquiries can be directed to the corresponding author.

## Ethics statement

The studies involving humans were approved by the ethics committee of the Maastricht University Medical Center (MUMC+). The studies were conducted in accordance with the local legislation and institutional requirements. The participants provided their written informed consent to participate in this study.

## Author contributions

BV: Conceptualization, Formal analysis, Investigation, Methodology, Visualization, Writing – original draft, Writing – review & editing. AS: Conceptualization, Formal analysis, Writing – original draft, Writing – review & editing. LL: Conceptualization, Formal analysis, Writing – original draft, Writing – review & editing. AT: Data curation, Methodology, Resources, Writing – review & editing. ED: Conceptualization, Supervision, Writing – review & editing. RB: Supervision, Writing – review & editing.
