# Supplementary material for: Practical tips by peer support in chronic vestibular hypofunction: an exploratory survey
Source: Front Neurol. 2024 Jan 3;14:1334038. doi: 10.3389/fneur.2023.1334038 (PMC10791824; doi:10.3389/fneur.2023.1334038)
Supplement: Supplementary file 1 [file Table_1.DOCX]

| **Tips and tricks from experience experts in balance issues in daily life** | |
| --- | --- |
| This is a voluntarily administered questionnaire. This survey is devised by the vestibular laboratory of Maastricht University Medical Centre (MUMC+).  As an experience expert, you possess a good understanding of the phenomenon of “living with vestibular impairment”. Through this survey, we would like to ask you about your tips and tricks you employ to enhance your quality of life and safety of daily activities.  We are looking forward to your invaluable contributions. Our aspiration is to improve our ways of advising potential patients to guide them in effectively managing their vestibular complaints. Your input could be integrated in patient-oriented resources (e.g. brochures, website content) within the otorhinolaryngology outpatient department and will be used for research purposes.   For illustrative purposes, a few examples are given below:   - Recommendation for walking: opting for spacious pedestrian pathways - Suggestions for household activities: Installation of supplementary handrails | |
| *Age (years)* |  |
| *Gender* |  |
| *Duration of balance symptoms (years)* |  |
| *Etiology of vestibular impairment (if known)* |  |
| *Severity of balance symptoms (0-10)* |  |
| **Please note your tips and tricks below** | |
|  | |
